# Impact of a rash management guide in patients receiving apalutamide for high-risk localized prostate cancer in the Apa-RP study

**DOI:** 10.1038/s41391-024-00858-4

**Published:** 2024-07-05

**Authors:** Neal Shore, Jason Hafron, Daniel Saltzstein, Amitabha Bhaumik, Pankaj Aggarwal, Jennifer Phillips, Tracy McGowan

**Affiliations:** 1 Carolina Urologic Research Center, Myrtle Beach, SC USA; 2 Michigan Institute of Urology, West Bloomfield, MI USA; 3 Urology San Antonio, San Antonio, TX USA; 4 Janssen Research and Development, Titusville, NJ USA; 5 Janssen Medical Affairs, Horsham, PA USA

**Keywords:** Prostate cancer, Cancer

## Abstract

**Background/objectives:**

Based on the SPARTAN and TITAN studies, apalutamide is approved for patients with nonmetastatic castration-resistant and metastatic castration-sensitive prostate cancer. Skin rash was a common adverse reaction across indications. We hypothesized that earlier identification and intervention could improve rash outcomes.

**Subjects/methods:**

A prespecified rash management guide outlining recommended skin care practices was provided to all patients enrolled in Apa-RP (NCT04523207). Rash-related safety data from Apa-RP were compared descriptively with data from SPARTAN and TITAN.

**Results:**

Patients in Apa-RP experienced improved rash-related outcomes vs those in SPARTAN and TITAN.

**Conclusions:**

Increased vigilance and proactive management may reduce the incidence, severity, and duration of rash during apalutamide treatment.

## Introduction

Apalutamide is a competitive antagonist of the androgen receptor [1, 2], approved for the treatment of patients with nonmetastatic castration-resistant prostate cancer and metastatic castration-sensitive prostate cancer [2] based on the results from two global Phase 3 trials: SPARTAN (nonmetastatic castration-resistant prostate cancer; NCT01946204) [3] and TITAN (metastatic castration-sensitive prostate cancer; NCT02489318) [4]. Skin rash is one of the more common adverse reactions observed with apalutamide treatment, occurring in approximately 25% of treated patients [5]. In SPARTAN and TITAN, rash was managed reactively with topical corticosteroids, oral antihistamines, and oral corticosteroids, and less frequently, with dose reduction and interruptions [5].

Based on the data collected in these earlier trials, a patient-empowered rash management guide was developed as a proactive approach to improve rash-related outcomes in the Apa-RP study (NCT04523207). Apa-RP was a multicenter, open-label, single-arm Phase 2 study of adjuvant apalutamide and androgen deprivation therapy (ADT) in treatment-naïve patients with high-risk localized prostate cancer who underwent radical prostatectomy (RP). The rationale for Apa-RP was based on the lack of effective treatment for this patient population, where patients are at increased risk of disease recurrence. We hypothesized that adjuvant apalutamide and ADT could improve biochemical recurrence-free survival in this patient population.

Here, we present an overview of the prespecified guide for rash management in Apa-RP and report rash-related safety data from Apa-RP compared descriptively with data from SPARTAN [3] and TITAN [4].

## Methods

**Fig. 1:**
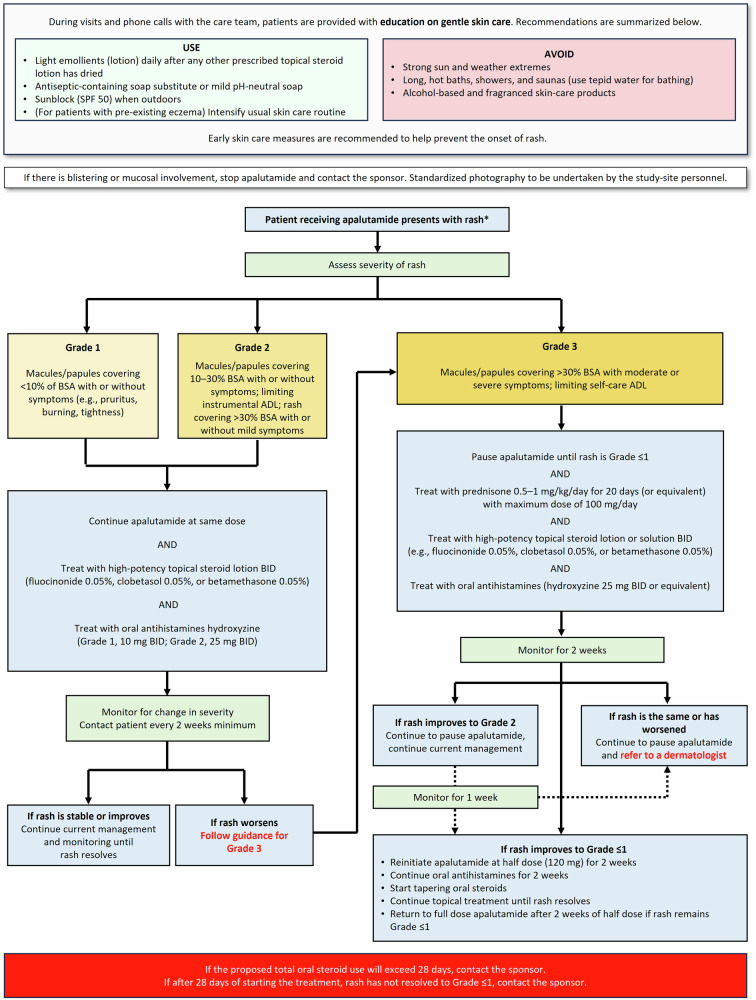
Schematic for prespecified rash management guide in Apa-RP. *Rash is a grouped term that includes MedDRA Preferred Terms related to the general term “rash.” ADL activities of daily living, BID twice daily, BSA body surface area, SPF sun protection factor.

In Apa-RP, the prespecified rash management guide outlined proactive steps to educate patients on gentle skin care to reduce the onset and severity of rash events, including the use of light emollients and antiseptic-containing soap substitutes. Patients were advised to avoid exposure to strong sunlight and hot water baths, showers, and saunas (Fig. 1). Institutional Review Board-approved printed handouts were provided to help educate patients in best practices. A standardized questionnaire was recommended during scheduled phone calls to help patients take preventive measures against and identify the onset of rash quickly, and to immediately seek necessary medical attention. The questionnaire included open-ended questions pertaining to onset, symptoms, severity, evolution, and treatment of rash. Calls were conducted by trained study personnel and were scheduled weekly in the first month of treatment, bi-weekly in months two and three, and as needed thereafter if the patient presented with rash. All site staff involved in the study were trained on rash management.

Rash events were graded per the National Cancer Institute Common Terminology Criteria for Adverse Events (NCI-CTCAE) Version 5.0, with a scale of grade 1 (least severe) to grade 3 (most severe) based on percentage of involved body surface area, presence, and severity of related symptoms (e.g., pruritus, burning, tightness), and impact on activities of daily living. Study sites were provided with a rash management guide containing the recommended procedure for managing rash (summarized in Fig. 1). Incidence of rash was reported by the patient, caregiver, surrogate, or legal representative, and summarized descriptively.

Apa-RP included an exclusively US population recruited from 28 US community urologic practices. Data on rash incidence, severity, treatment, time to first onset, resolution, and time to resolution during the first 12 months following initiation of apalutamide treatment were collected and compared with data from SPARTAN [3] and TITAN [4]. To mitigate potential bias stemming from variations in rash incidence among different geographical populations [5–7], only data from North American populations of SPARTAN and TITAN were included in this comparison. All patients enrolled in Apa-RP were included in the rash comparison.

Time to first onset of rash was defined as the number of days from first dose of the study drug to first rash. Rash was considered resolved on resolution of all skin lesions. Time to resolution was defined as the number of days from the date of first onset of rash to complete resolution of rash.

## Results

All 108 patients enrolled in Apa-RP were treated with apalutamide 240 mg once daily and ADT for 12 cycles (28-day cycles). Fewer patients experienced any-grade rash in Apa-RP vs those in the North American populations of SPARTAN and TITAN (21.3% vs 28.3% and 33.3%, respectively) (Table 1). Over 60% of rash events in Apa-RP were grade 1, compared with 40.0% and 28.6% in the North American populations of SPARTAN and TITAN. Median time to first report of rash was shorter in Apa-RP at 72.0 days, vs. SPARTAN (97.5 days) and TITAN (84.0 days). Median time to rash resolution was also shorter in Apa-RP at 49.0 days, vs SPARTAN (60.0 days) and TITAN (142.0 days). The proportion of patients with any-grade rash who underwent dose reductions was lower in Apa-RP (4.3%) vs SPARTAN (10.0%) and TITAN (19.0%) (Table 1). The proportion of patients with any-grade rash who discontinued treatment was 0% in Apa-RP, vs 6.3% in SPARTAN and 9.5% in TITAN (Table 1). The proportion of patients who developed rash and received systemic corticosteroids, topical corticosteroids, or oral antihistamines varied between the three studies (Table 1).

**Table 1:** Side-by-Side comparison of rash-related data from Apa-RP, SPARTAN, and TITAN (North American safety populations).

	Apa-RP (*N* = 108)	SPARTAN (*N* = 283)	TITAN (*N* = 63)
Treatment-emergent rash^a^ in apalutamide-treated groups			
Worst skin rash grade during study, *N* (%)	23 (21.3)	80 (28.3)	21 (33.3)
Grade 1, *n/N* (%)^b^	14/23 (60.9)	32/80 (40.0)	6/21 (28.6)
Grade 2, *n/N* (%)^b^	6/23 (26.1)	30/80 (37.5)	8/21 (38.1)
Grade 3, *n/N* (%)^b^	3/23 (13.0)	18/80 (22.5)	7/21 (33.3)
Median time to onset of first skin rash, days (range)^c^	72.0 (10.0–246.0)	97.5 (1–1301)	84.0 (9–394)
Resolved, *n/N* (%)^b,d^	22/23 (95.7)	75/80 (93.8)	12/21 (57.1)
Median time to resolution, days (range)	49.0 (4–290)	60.0 (1–1529)	142.0 (4–538)
Dose reduction of study drug following onset of treatment-emergent rash, *n/N* (%)^b^	1/23 (4.3)	8/80 (10.0)	4/21 (19.0)
Treatment interruption following onset of treatment-emergent rash, *n/N* (%)^b^	5/23 (21.7)	19/80 (23.8)	7/21 (33.3)
Discontinuation of study drug following onset of treatment-emergent rash, *n/N* (%)^b^	0	5/80 (6.3)	2/21 (9.5)
Received any treatment for rash, *n/N* (%)^b^	12/23 (52.2)	36/80 (45.0)	11/21 (52.4)
Received topical steroid, *n/N* (%)^b^	12/23 (52.2)	21/80 (26.3)	11/21 (52.4)
Received antihistamine, *n/N* (%)^b^	7/23 (30.4)	22/80 (27.5)	2/21 (9.5)
Received systemic steroid, *n/N* (%)^b^	4/23 (17.4)	17/80 (21.3)	3/21 (14.3)

^a^Rash is a grouped term which includes MedDRA Preferred Terms related to the general term “rash.”

^b^Percentage is presented as proportion of patients with any grade rash.

^c^Regardless of grade of first skin rash. The starting time for median calculation is the date of the first dose of the study drug.

^d^Resolved was defined as all skin rashes being reported as resolved (regardless of initial or worst grade). The starting time for median calculation is the start date of the first skin rash.

## Discussion

The rash management guide developed for Apa-RP provides a proactive, patient-empowered approach to monitoring and managing rash events. In relation to rash, patients in Apa-RP experienced lower overall incidence, reduced severity, a shortened time to resolution, and lower rates of dose reduction and treatment interruption or discontinuation, vs those in SPARTAN and TITAN. Notably, no patients in Apa-RP required treatment discontinuation due to rash. The relative improvements in rash management in Apa-RP suggest that early identification and intervention may help to reduce the incidence, severity, and time to resolution of rash during treatment with apalutamide. The preventative measures presented in the Apa-RP rash management protocol may have contributed to the lower incidence of rash compared with SPARTAN and TITAN.

This study is limited by the small number of patients (*N* = 108) included only from North America. Previous studies have reported that the incidence of rash following apalutamide treatment varies across different geographical populations (for example, a higher incidence was reported among East Asian patients compared with the global population) [5–7]. Therefore, the analysis presented in this report only included participants from the North American populations of SPARTAN and TITAN to mitigate any potential bias.

While SPARTAN and TITAN studies recorded rash data beyond 12 months following initiation of apalutamide treatment, most patients (> 80%) experienced onset of rash within the first 12 months of treatment (Supplementary Tables 1 and 2). Data for dose modification or discontinuation beyond 12 months were not recorded for either study.

Although the patients included in this analysis span three prostate cancer disease states, the populations were considered similarly susceptible to rash, patients were receiving the same dose of apalutamide with ADT, and the same rash-related endpoints were recorded. Additionally, data were compared descriptively, with no comparative statistical analyses applied.

In conclusion, these findings suggest that increased vigilance from the care team and empowering patients through education may reduce the incidence, duration, and severity of rash during apalutamide treatment. Incorporating a proactive rash management guide into clinical practice appears to be warranted based on the data presented.

## Supplementary information

Supplementary Table 1

Supplementary Table 2

## DATA SHARING

The data sharing policy of Janssen Pharmaceutical Companies of Johnson & Johnson is available at https://www.janssen.com/clinical-trials/transparency. As noted on this site, requests for access to the study data can be submitted through Yale Open Data Access [YODA] Project site at http://yoda.yale.edu.
