# Supplementary material for: Impact of a rash management guide in patients receiving apalutamide for high-risk localized prostate cancer in the Apa-RP study
Source: Prostate Cancer Prostatic Dis. 2024 Jul 5;28(3):828–31. doi: 10.1038/s41391-024-00858-4 (PMC12399413; doi:10.1038/s41391-024-00858-4)
Supplement: Supplementary file 1 — Supplementary Table 1 [file 41391_2024_858_MOESM1_ESM.rtf]

Supplementary Table 1:	Patients with treatment-emergent adverse events of rash and time to onset
of first skin rash in apalutamide-treated arm of SPARTAN (NCT01946204) – Safety population	
	Apalutamide 	
Analysis set: Safety Analysis Set	803	
		
Subjects with TE Rash	212 (26.4%)	
		
Time (Months) to Onset of First Skin Rash		
N	212	
≤ 12 Months	174 (82.1%)	
12 – ≤ 24 Months	24 (11.3%)	
> 24 Months	14 (6.6%)	
		
Time (Months) to Onset of First Skin Rash		
N	212	
Mean (SD)	6.58 (8.576)	
Median	3.06	
Range	(0.0; 42.7)	

Note: Treatment-emergent adverse events are those that occurred between the date of 1st dose of study drug and date of last dose of study drug plus 30 days.
Note: Adverse events are coded using Medical Dictionary for Regulatory Activities Version 19.1.	
	
SD, standard deviation; TE, treatment-emergent.
